# Nursing education research in Sub-Saharan Africa: A systematic review and bibliometric analysis

**DOI:** 10.1371/journal.pdig.0000900

**Published:** 2025-06-26

**Authors:** Beth Waweru, Peter Gatiti, Serah Wachira

**Affiliations:** 1 School of Nursing and Midwifery, Aga Khan university, Nairobi, Kenya; 2 Faculty of Health Sciences, Aga Khan university, Nairobi, Kenya; 3 School of Nursing and Midwifery, Aga Khan university, Nairobi, Kenya; Fundación Progreso y Salud: Junta de Andalucia Consejeria de Salud y Familias Fundacion Progreso y Salud, SPAIN

## Abstract

Nursing education is pivotal for ensuring competent healthcare professionals, and its improvement is essential for enhancing the quality of health care systems globally. This study focuses on nursing education research in Sub-Saharan Africa (SSA) over the last decade, employing both bibliometric analysis and systematic review methodologies. The bibliometric analysis reveals an evolving landscape of nursing education research in SSA, offering insights into trends, key countries, journals, and predominant research themes. Notably, the study identifies a scarcity of literature using bibliometric approaches in nursing research, addressing this gap by providing a comprehensive overview of the field.The systematic review, guided by the Preferred Reporting Items for Systematic Reviews and Meta-Analyses (PRISMA) guidelines, explores 1359 articles published in the last ten years, focusing on nursing education in SSA. The analysis of 1288 selected articles emphasize experiences and challenges faced by nursing and midwifery students during their education and clinical training. The emerging themes cuts across classroom teaching, clinical learning environments, and overall clinical practice. The findings highlight the need for attention to educational support, effective communication, professionalism, inclusivity, and innovative teaching methods. Limitations include the exclusive focus on SSA, restricting generalizability to other regions. Nonetheless, the study offers valuable insights for educators, policymakers, and institutions to enhance the quality of nursing education. By addressing identified challenges, fostering innovation, and promoting inclusivity, stakeholders can better prepare students to meet the dynamic demands of the healthcare profession in SSA and potentially other regions, especially Low- and Middle-income Countries. The research contributes to the ongoing efforts to bridge the gap between nursing education theory and practice, ultimately improving healthcare outcomes in the region.

## Author summary

Nursing education is essential for strengthening healthcare systems, yet research on this topic in Sub-Saharan Africa (SSA) remains fragmented. This bibliometric study analyzed the trends, impact, and collaboration patterns in nursing education research across the region. By examining publications over the past decades, we identified key research themes, influential authors, and the countries contributing the most to this field. The findings highlight gaps in research productivity, limited regional collaborations, and the need for more locally driven studies. This study provides valuable insights for policymakers, educators, and researchers aiming to advance nursing education and improve healthcare outcomes in SSA.

## 1 Introduction

Nursing education is a key component of the nursing profession. Nurses have to be prepared well theoretically for them to be able to translate the acquired knowledge to practice. Production of nurses and midwives with relevant competencies remains a critical role in nursing education and its improvement enhances the quality of health care and health systems [1]. Nursing education research is a great contributor of evidence based practice globally [2]. The main focus of research in nursing education is on improving outcome of patient care through evidence based training [3]. Contextualization of nursing research helps to identify the key issues that are specific to the context as well as inform policy and practice.

Nursing education research in Sub-Saharan Africa (SSA) contribute to a better understanding of the challenges and opportunities associated with nursing education in the region. Nursing education research has gained considerable attention in SSA and the major topics in the last 10 years include; clinical simulation, curriculum, teaching strategies, interprofessional education, role development and competencies, advances and specialization and innovations.

In particular, curriculum development research has concentrated on specialization courses to enhance the role of the nurse in resource constrained settings [4–6]. The main areas of specialization include pediatric nursing, critical care and emergency nursing. Teaching and learning strategies have also been evaluated to embrace 21st century pedagogies. For example, collaborative learning simulation [7], use of technology in teaching and learning [8], as well as gaming [9] to improve skills competency. The use of technology in nursing education for teaching and learning has also been explored especially the evolving role of the nurse in improving health care outcomes [10].

An analysis of the aspects of research is helpful in linking the contextualized research that can help countries in SSA make curriculum decisions for improvement in training outcomes [11]. A clear understanding of trends in nursing education research can trigger more research output in SSA by highlighting gaps in research that have not been explored [12].

One of the techniques that has been used to examine nursing research globally is bibliometric analysis. Bibliometric analysis involves using quantitative analytical methods to assess the output of academic literature, outlining the major research areas, the core journals, article output, country of origin, authors, and most searched keywords [13]. However, there is paucity of nursing research using bibliometric approach [14]. Therefore, this study provides an alternative approach to nursing research through using bibliometric approaches to examine nursing education research in SSA.

The incorporation of systematic review into the bibliometric analysis plays a pivotal role in advancing nursing education research in Sub-Saharan Africa by offering a comprehensive and evidence-based approach to understanding the current state of knowledge [15]. They consolidate existing research findings, enabling educators and policymakers to make informed decisions that can improve the quality of nursing education in the region. The systematic review aspect of this research will help identify what is known, synthesizing data from various sources to provide a clear picture of the current evidence, which is especially crucial in a resource-constrained setting like Sub-Saharan Africa [16]. Additionally, the review pinpoints gaps in the existing literature, shedding light on areas where further research is needed [17]. In the context of nursing education, this is invaluable, as it not only directs future research efforts but also guides the development of curricula and teaching strategies tailored to the unique needs of Sub-Saharan Africa.

This study attempted to answer the following research questions

RQ1: How has nursing education research in SSA evolved over time?
RQ2: What are the main countries and key journals in nursing education research across SSA
RQ3: Which areas of nursing education research are more predominant across the SSA countries
RQ4: What are the various themes that the literature on nursing education in SSA revolves around?

## 2 Methodology

This study adopted bibliometric analysis and content analysis to provide a comprehensive overview of the extant literature on nursing education research. Various researchers have used Scopus database to undertake bibliometric analysis [18,19]. SCOPUS is the largest abstract and citation database of peer-reviewed literature. The data for this study were extracted from SCOPUS and the category chosen for analysis was “nursing education”. The results were exported to Microsoft excel for further graphical representation.

**Fig 1:**
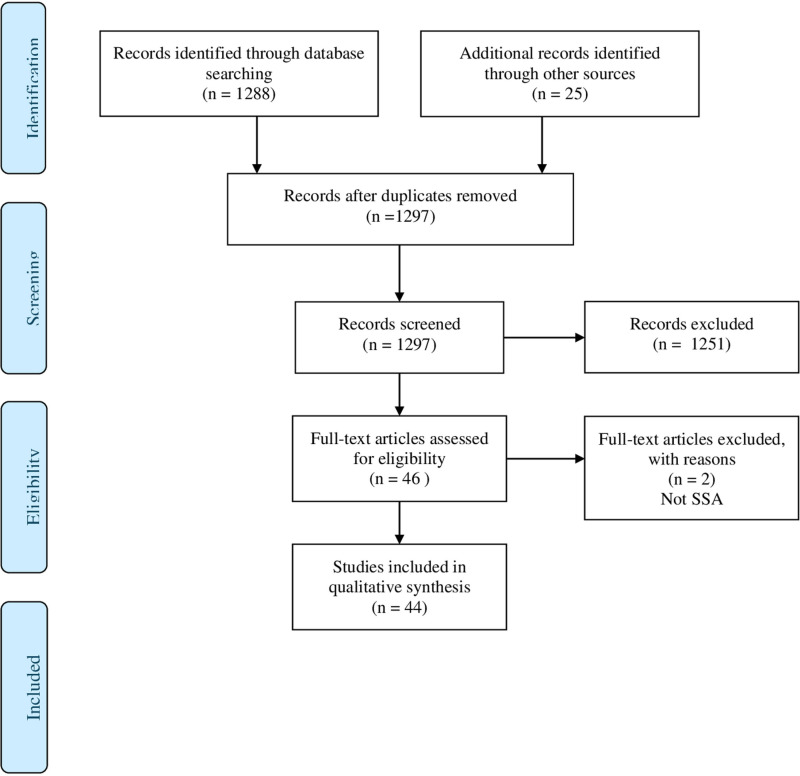
PRISMA Flow Diagram.

The preferred reporting items for systematic reviews and meta-analyses (PRISMA) flow diagram was used to collect data for the systematic literature review [20]. The PRISMA statement consists of a four-phase flow diagram as shown in Fig 1. The use of the PRISMA flow diagram improves the quality of literature review as it provides guidelines that ensure reviewed studies are reported comprehensively and in a transparent manner [21].

### 2.1 Exclusion criteria

- Articles published in languages other than English
- Systematic reviews/literature reviews and other review articles
- Studies undertaken in other geographical locations other that Sub Saharan Africa
- Studies undertaken before 2013 and after 2022
- Studies not focusing on “nursing education” OR “Nursing education research”

The number of articles retrieved from the search string was 1359. We limited our search to articles published in English in the last ten years (2013–2022), and a total of 1170 articles and 118 reviews were retrieved. To ensure the study’s quality, we only included journal articles and reviews. We did not include conference papers, notes, and book chapters. For the bibliometric analysis, 1288 articles were included in the final dataset.

### 2.2 Study selection and data collection

The first screening was based on a double-screening of titles and abstracts by two independent reviewers. Results which met explicit exclusion criteria were excluded. The remaining manuscripts were assessed for full-text reading. In case of disagreement among reviewers, a third reviewer assessed the study and a decision for inclusion was reached by consensus. Data was entered in a spreadsheet.

The researchers carried out a qualitative synthesis of the results from the included studies. Details of the search process are shown in Fig 1 and Table 1 below.

**Table 1:** Databases searched and search queries.

Date	Database	Search Query	Hits
12/05/2023	SCOPUS	(KEY (“nursing education”) OR KEY (“nursing education research”)) AND PUBYEAR > 2012 AND PUBYEAR < 2023 AND (LIMIT-TO (LANGUAGE, “English”)) AND (LIMIT-TO (AFFILCOUNTRY, “South Africa”) OR LIMIT-TO (AFFILCOUNTRY, “Ghana”) OR LIMIT-TO (AFFILCOUNTRY, “Malawi”) OR LIMIT-TO (AFFILCOUNTRY, “Nigeria”) OR LIMIT-TO (AFFILCOUNTRY, “Kenya”) OR LIMIT-TO (AFFILCOUNTRY, “Tanzania”) OR LIMIT-TO (AFFILCOUNTRY, “Uganda”) OR LIMIT-TO (AFFILCOUNTRY, “Ethiopia”) OR LIMIT-TO (AFFILCOUNTRY, “Rwanda”) OR LIMIT-TO (AFFILCOUNTRY, “Botswana”) OR LIMIT-TO (AFFILCOUNTRY, “Namibia”) OR LIMIT-TO (AFFILCOUNTRY, “Zambia”) OR LIMIT-TO (AFFILCOUNTRY, “Zimbabwe”) OR LIMIT-TO (AFFILCOUNTRY, “Sierra Leone”) OR LIMIT-TO (AFFILCOUNTRY, “Cameroon”) OR LIMIT-TO (AFFILCOUNTRY, “Liberia”) OR LIMIT-TO (AFFILCOUNTRY, “Lesotho”) OR LIMIT-TO (AFFILCOUNTRY, “Somalia”) OR LIMIT-TO (AFFILCOUNTRY, “Burkina Faso”) OR LIMIT-TO (AFFILCOUNTRY, “Congo”) OR LIMIT-TO (AFFILCOUNTRY, “Gambia”) OR LIMIT-TO (AFFILCOUNTRY, “Mozambique”) OR LIMIT-TO (AFFILCOUNTRY, “Sudan”) OR LIMIT-TO (AFFILCOUNTRY, “Togo”) OR LIMIT-TO (AFFILCOUNTRY, “Guinea”) OR LIMIT-TO (AFFILCOUNTRY, “Senegal”) OR LIMIT-TO (AFFILCOUNTRY, “Mali”) OR LIMIT-TO (AFFILCOUNTRY, “Angola”) OR LIMIT-TO (AFFILCOUNTRY, “Chad”) OR LIMIT-TO (AFFILCOUNTRY, “Eritrea”) OR LIMIT-TO (AFFILCOUNTRY, “Madagascar”))	586
12/05/2023	PubMed	(“education, nursing”[MeSH Terms] AND “Nursing Education Research”[MeSH Terms] AND “Africa South of the Sahara”[MeSH Terms]) AND ((english[Filter]) AND (2013:2022[pdat]))	24
12/05/2023	CINAHL	“education, nursing” AND “Nursing Education Research” AND “Africa South of the Sahara” AND ((english[Filter]) AND (2013:2022[pdat]))	82
12/05/2023	AJOL	“nursing education” OR “nursing education research” in Sub-Saharan Africa AND ((English) AND (2013:2022)	133
12/05/2023	CORE	“nursing education” OR “nursing education research” in Sub-Saharan Africa AND ((English) AND (2013:2022)	12063

## 3 Descriptive analysis

To answer the first and second research questions, a descriptive analysis was carried out to determine the publication trend of the scientific articles as well as identify the top countries, journals and institutions actively involved in nursing education research.

### 3.1 Publication output

**Fig 2:**
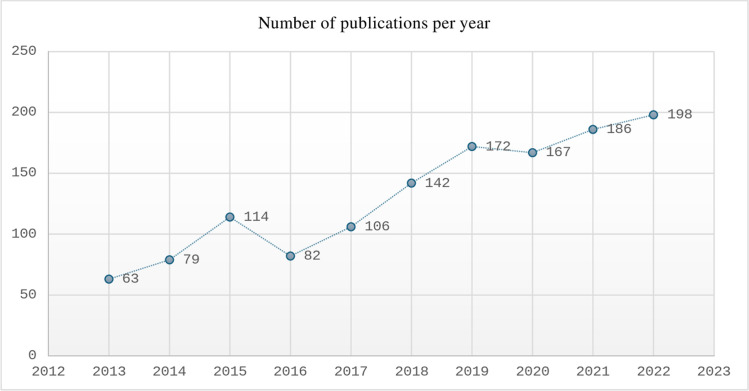
Publication trend during past 10 years.

Fig 2 shows that the number of articles on nursing education research grew from 63 articles in 2013–195 articles in 2022. The significant growth reflects the interest of researchers in the field of nursing education. This trend indicates that nursing education research will continue to rise in the coming years.

### 3.2 Nursing education research publications by country

**Fig 3:**
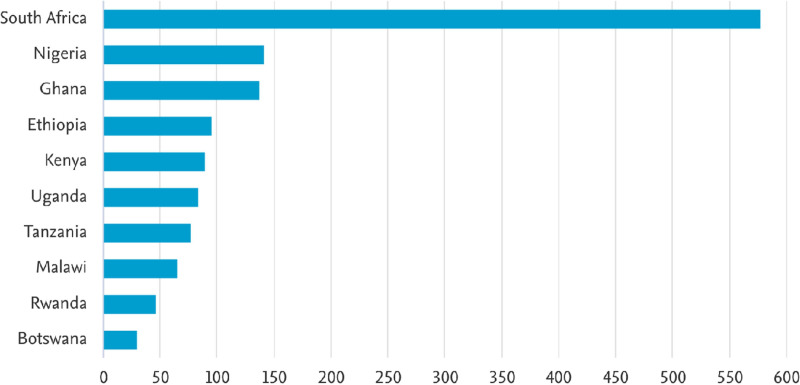
Publications country wise.

Fig 3 shows the top 10 countries within SSA which are actively involved in nursing education research. As shown in Fig 3, the majority of the articles were published by researchers from

South Africa (566 articles), followed by Nigeria (139 articles), Ghana (135 articles), Ethiopia (94 articles) and Kenya (86 articles).

### 3.3 Nursing education research top institutions

**Fig 4:**
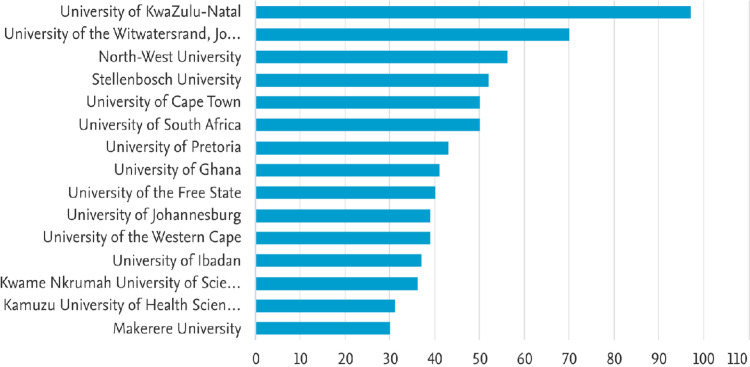
Nursing education research top 15 institutions.

The top 5 institutions actively involved in nursing education research are University of KwaZulu-Natal (95 publications), University of the Witwatersrand (69 publications), North-West University (55 publications), Stellenbosch University (51 publications), and University of Cape Town (48 publications) as depicted in Fig 4. It is noteworthy that all the top five institutions are from South Africa. Among the top 15 institutions, three institutions were from West Africa (University of Ghana, University of Ibadan, and Kwame Nkrumah University of Science and Technology). It is interesting to note that only one institution from East Africa made it to the top 15 list (Makerere University).

### 3.4 Nursing education research subject areas

**Fig 5:**
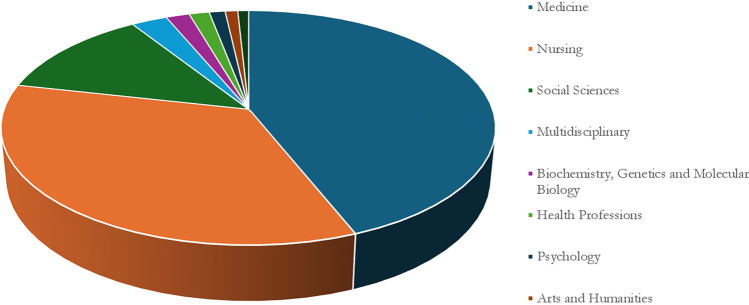
Distribution of publications in various subjects.

As indicated in Fig 5, nursing education research represents different subject areas and disciplines. Medicine was the most significant field with the highest number of articles (682) followed by Nursing (537), Social Sciences (193), Multidisciplinary (43), and Biochemistry, Genetics and Molecular Biology (27).

### 3.5 Nursing education research publications by source

**Fig 6:**
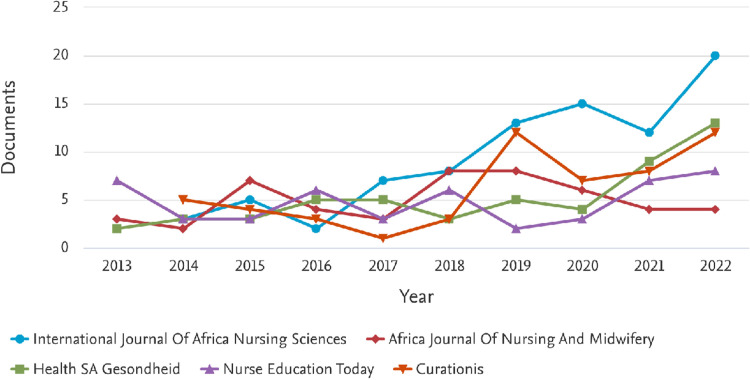
Nursing education most productive journals.

Nursing education researchers from SSA publish in a wide range of journals. The scatter across journals is striking. As demonstrated in Fig 6, the top 5 journals where the majority of nursing education articles were published are the International Journal of Africa Nursing Sciences (85 articles), Curationis (55 articles), Health SA Gesondheid (52 articles), Nurse Education Today (48 articles), and Africa Journal of Nursing and Midwifery (45).

### 3.6 Nursing education research: analysis by funding sponsors

**Fig 7:**
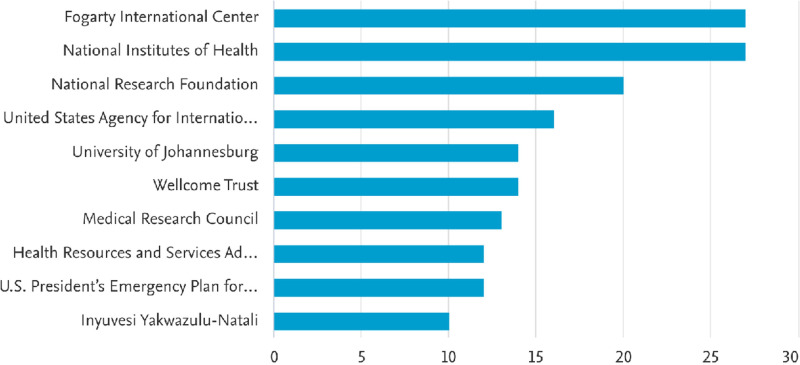
Nursing education research funding sponsors.

Nursing Education Research in SSA is funded by a variety of organisations. As demonstrated in Fig 7, the top funding organisations are Fogarty International Center (16.4%), National Institutes of Health (16.4%), National Research Foundation (12.1%), United States Agency for International Development (9.7%), Wellcome Trust (8.5%). and University of Johannesburg (8.5%).

### 3.7 Societal impact of nursing education research from SSA

Altmetric explorer was used to measure the societal impact and the societal attention of nursing education research in SSA as depicted in Table 2. Altmetrics (alternative metric) is a tool that can help identify online activity in order to demonstrate research impact. It measures research impact by providing information on web-driven scholarly interactions for an article and is intended to be complementary to traditional, citation-based metrics [22]. The top 10 highly cited nursing education in SSA research papers have been mentioned in 9 policy documents including 3 mentions by World Health Organization, and 1 mention each by World Bank, CDC, European Union, Norwegian Institute of Public Health, Australian Department of Health, and Institute of Network Cultures.

**Table 2:** Altmetric analysis of top 10 highly cited papers.

#	Title	Citations	Attention Score	Tweets
1	Informal mobile learning in nurse education and practice in remote areas-A case study from rural South Africa	56	10	9
2	Barriers and facilitators to the implementation of lay health worker programmes to improve access to maternal and child health: Qualitative evidence synthesis	332	72	73
3	Barriers Associated With Evidence-Based Practice Among Nurses in Low- and Middle-Income Countries: A Systematic Review	68	4	6
4	Reducing HIV-related stigma and discrimination in healthcare settings: A systematic review of quantitative evidence	65	6	8
5	Facilitating problem-based learning among undergraduate nursing students: A qualitative systematic review	63	74	110
6	Evaluation of Helping Babies Breathe Quality Improvement Cycle (HBB-QIC) on retention of neonatal resuscitation skills six months after training in Nepal	63	3	3
7	Nursing education challenges and solutions in Sub Saharan Africa: An integrative review	84	57	7
8	How to achieve the desired outcomes of advance care planning in nursing homes: A theory of change	61	31	28
9	Barriers to providing quality emergency obstetric care in Addis Ababa, Ethiopia: Healthcare providers’ perspectives on training, referrals and supervision, a mixed methods study	65	2	1
10	How well does pre-service education prepare midwives for practice: Competence assessment of midwifery students at the point of graduation in Ethiopia	58	6	7

## 4 Bibliometric analysis

To answer the third research question, we carried a bibliometric analysis through conducting a citation analysis and co-occurrence of keywords analysis to determine the main research areas

related to nursing education research. Bibliometric analysis is a rigorous method used to explore and analyze large volumes of scientific data to unpack the evolutionary nuances of a specific field and shed light on the emerging areas in that field [23].

The researchers carried out a citation analysis and performed co-occurrence of keywords analysis using the VoS viewer software to identify the core areas of research as well as the emerging topics associated with nursing education research. The findings serve as the roadmap for future researchers, interested in undertaking research in nursing education.

### 4.1 Citation analysis of nursing education research

**Fig 8:**
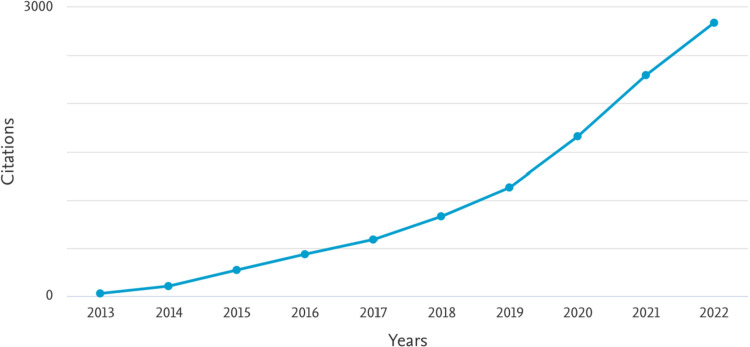
Citation analysis of nursing education research.

Nursing education research articles were cited 10,176 times from 2013 to 2022. Fig 8 shows the citation overview of the 1288 documents over a period of ten years. Findings indicate that there was an exponential growth in nursing education research from 2013 (27 citations) to 2022 (2,823 citations).

### 4.2 Co-occurrence of keywords analysis

**Fig 9:**
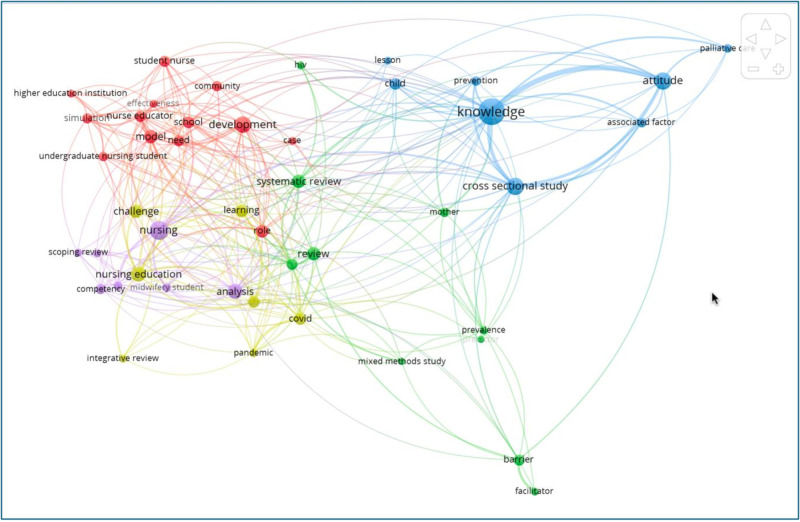
Co-occurrence analysis of author keywords.

Through VOS viewer software, we identified the most popular keywords used in nursing education research. The findings demonstrate that following are the most used keywords: barriers, facilitators, attitude, development, knowledge, competency, and challenges (Fig 9).

The author keywords in yellow colour demonstrates how recently they have been used in the existing literature. It is evident that challenge, learning, and Covid are the most emerging topics in the nursing education literature.

## 5. Discussion of the main themes in the literature

To answer the fourth research question, we classified the published literature and divided it into 6 research clusters. These research streams are the main areas that have received much attention in the past ten years. The major focus areas are: nursing education barriers and facilitators, knowledge and attitude of nurses towards nursing education.

### 5.1 Nursing education barriers and facilitators

The study retrieved 8 studies that considered challenges associated with nursing education. Table 3 contains a summary of key findings of the challenges.

**Table 3:** Nursing education challenges (n = 8).

Study	Country	Study Design	Main Findings
` [1]	All SSA	Integrative review	Sub Saharan Africa needs more reforms to increase capacity of educators and mentors. The region also needs strong regulatory frameworks, availability of infrastructure and adequate resources.
[24]	All SSA	Narrative literature review	Challenges included provision of adequate resources, monitoring of academic dishonesty, provision of technical support and revision of the curriculum.
[25]	Malawi	Phenomenological study	Key challenges include failure to fulfil the clinical teaching requirements, and attitude of nurse educators towards students.
[26]	South Africa	Exploratory	Main challenges experienced by pregnant student nurses were academic, failure to write examinations, support system and maternity leave.
[27]	South Africa	Case study	Challenges included poor implementation of the peer-group clinical mentoring programme, ineffective programmes, poor attitude of the mentors, unprofessional conduct and communication challenges.
[28]	South Africa	Case study	Lack of time, lack of incentives, and lack of mentors to learners through the complex process of publishing
[29]	Ghana	Descriptive	Challenges faced by nurse educators in low-resource settings include environmental factors, institutional challenges, and weak regulatory frameworks.
[30]	South Africa	Descriptive	Raising awareness of nurse educators assists them in becoming more student-centred in their teaching.

It is clear from the research that nursing education in Sub-Saharan Africa faces a range of challenges. These challenges have implications for both the quality of education and the preparedness of nursing students to provide quality healthcare.

The key challenges identified include lack of capacity development [1], regulatory challenges [29], and resource disparities in low and middle-income countries [28].

Some challenges are specific to learning. For example, [24] highlighted issues related to the transition to online learning in nursing education. [26] identified educational challenges for pregnant student which include difficulty in writing examinations, a lack of support systems, and maternity leave concerns, which can disrupt their education. [27] highlighted challenges in peer mentoring programs for nursing students.

Other challenges involve clinical placement. [25] noted that challenges related to fulfilling clinical teaching requirements and negative attitudes of nurse educators can hinder students’ learning experiences during these placements. Similarly, [30] identified challenges faced by nurse educators in teaching clinical reasoning skills. These challenges can impact students’ ability to make sound clinical judgments.

Addressing these challenges is crucial for improving nursing education and ensuring that nursing students are adequately prepared to provide high-quality healthcare. This may involve policy changes, increased investment in education infrastructure, faculty development, and the adoption of innovative teaching methods, among other strategies.

Nursing education research in Sub-Saharan Africa (SSA) has steadily grown, with South Africa, Nigeria, and Ghana leading in research output (Fig 3). While this progress is encouraging, it also highlights significant disparities in regional research capacity. Many countries still lack the necessary resources and institutional support to generate evidence-based strategies for curriculum development, faculty training, and student learning support. One major factor influencing research capacity is funding. Organizations like the Fogarty International Center (16.4%), the National Institutes of Health (16.4%), and the National Research Foundation (12.1%) (Fig 7) play a crucial role in supporting nursing education research in SSA. However, the heavy reliance on external funding raises concerns about long-term sustainability. Without more substantial regional investment in nursing research, SSA institutions may struggle to produce localized evidence that can inform policies and improve nursing education in a contextually relevant way. Beyond funding, nursing education in SSA faces persistent challenges such as faculty shortages, inadequate infrastructure, and limited access to innovative teaching methods [1,29] Many studies emphasize the importance of simulation-based education in enhancing clinical training [31]. However, resource constraints and a lack of faculty training hinder its full implementation [32]. Addressing these challenges will require deliberate investment in faculty development, digital learning tools, and stronger institutional collaborations to bridge the gap between research findings and real-world improvements in nursing education quality.

#### Bridging the gaps: strengthening collaboration in SSA.

While the volume of nursing education research in SSA is increasing, critical gaps remain in key areas such as innovative teaching methods, faculty development, student support systems, and digital health integration. Additionally, interprofessional education, competency-based training, and curriculum standardization remain underexplored (Fig 9). This lack of focus on transformative educational models limits the ability of SSA institutions to adapt to evolving healthcare needs.

To bridge these gaps, SSA institutions must leverage regional and international partnerships to optimize resources and strengthen research capacity. A three-pronged approach can be particularly effective:

1. Strengthening South-South collaboration
  - Joint research initiatives and faculty exchanges: Encouraging cross-country collaboration among SSA institutions to share best practices in nursing education research and teaching methods.
  - Regional education networks: Establishing standardized nursing curricula to improve student and faculty mobility across SSA institutions.
  - Shared simulation centers: Pooling resources to create regional simulation hubs where multiple institutions can access high-quality clinical training.
2. Expanding North-South partnerships
  - Mentorship and capacity building: Partnering with high-income country institutions to provide mentorship programs, funding access, and research training.
  - Co-publication and research visibility: Encouraging collaborative publishing between SSA and international researchers to increase global recognition of SSA nursing education research [33].
3. Leveraging digital networking and Open-Access knowledge platforms
  - Regional research repositories: Establishing open-access platforms where SSA researchers can share findings, best practices, and policy recommendations.
  - Virtual learning and digital mentorship: Using technology-driven solutions to connect SSA nursing educators with international nursing education experts, providing ongoing professional development opportunities [34].

By adopting these strategies, SSA institutions can build a stronger, more interconnected research ecosystem that fosters innovation, improves nursing education, and ultimately strengthens healthcare delivery across the region.

### 5.2 Experiences of nurses and nursing students on nursing education research

The study retrieved 16 studies that considered the experiences of nurses and nursing students on nursing education research. Table 4 contains a summary of key findings of their experiences.

**Table 4:** Experiences of nurses and nursing students on nursing education research (n = 16).

Study	Country	Study Design	Main Findings
[35]	Ghana	Descriptive cross-sectional study	• Students mostly learn in the clinical setting through observation and imitation. • Students do not always get the needed support in achieving their learning. objectives.
[36]	Ghana	Exploratory descriptive qualitative design	• Pre-examination conference between students and examiners help lessen students’ anxiety. • Quality of the assessment is affected by limited resources
[37]	Namibia	Qualitative, explorative study	• For effective learning to take place in ODL, timely and helpful feedback from tutors should be provided.
[37]	Namibia	Qualitative descriptive enquiry	• Need for well-articulated plans and actions from students, clinical instructors, lecturers, faculty management teams, and the nurses in practice facilities.
[38]	Cameroon	Qualitative design	• The use of a variety of interactive educational strategies is considered by nursing students as vital to enhancing learning.
[39]	South Africa	Qualitative, exploratory design	• Students experienced issues with role modelling, language barriers, and prejudice. • Unprofessional behaviour contribute negatively to the image of the profession.
[40]	Tanzania and Madagascar	Descriptive and convergent mixed method design	• Nursing students were satisfied with simulation as a pedagogic method, as it improved their competence and prepared them for professional practice.
[41]	Ghana	Descriptive exploratory qualitative approach	• Factors that influence clinical teaching skills: student characteristics, educator characteristics, cultural factors technology, and infrastructure.
[42]	Malawi	Qualitative descriptive design	• Feeling of being inferior, lack of recognition by some female-nurse midwives, and discrimination. • Lack of information regarding nursing prior to joining the profession. • Recommendations include career guidance
[43]	South Africa	Case study	• Main purpose of collaboration not achieved due to the lack of a common understanding of the concept of collaboration, lack of readiness to collaborate and a lack of sharing of resources.
[44]	Soth Africa	Quantitative study- Self reporting questionnaire	• Preventive actions are required to prevent and control violence towards students. • Informing students about violence, communicative skills, reaction, and coping with violence during studying is necessary.
[45]	South Africa	A qualitative, exploratory descriptive design	• Poor interpersonal skills, poor communication skills, anxiety, neglect, discrimination, congestion of students, inability to reach objectives, violence
[46]	Mauritius	A qualitative, exploratory, descriptive approach	• The mentoring practice was informal with unclear role expectations. • Poor material and personal resources further compounded the challenges.
[47]	Malawi	quantitative research design	• The roles of preceptors should involve facilitating students’ clinical teaching and learning. • Preceptors with more years of post•registration experience are less confident in their preceptorship role performance
[25]	Malawi	Hermeneutic phenomenological study	• Emotional labour was evident in students’ narrative accounts about their caring encounters, death and dying and caring.
[32]	South Africa	A descriptive quantitative research design	• Nurse educators had not been exposed to, and had limited experience utilising HFS as a teaching method • Facilities equipped with HFS manikins are not used optimally due to ‘fear of the unknown’ experienced by the nurse educators because of their lack of training, and/or the lack of technological skills

Newly graduated nurses face challenges such as inadequate supervision, lack of the clinical sites’ knowledge about program objectives [48]. Furthermore, issues related to resources, practice, and assessment have also been identified as challenges in nursing student education in Africa [36,37]. The utilization of portfolios as assessment tools brought forward themes of positive and negative experiences and the need for better communication and action plans among students, clinical instructors, and faculty management teams [37].

Recommendations emanating from clinical learning experiences have hence emphasized the need for more support for students to achieve educational objectives [35,45]. Undergraduate nursing students also encounter issues with professionalism, role modeling, language barriers, and their understanding of professional behavior [39]. Need for gender-inclusive communication strategies and the use of male nurse role models to encourage male student-nurse midwives have been emphasized [42]. A rare but important issue experienced by students is violence and emotional labour during their clinical learning experiences [25,44]. This underscores the importance of promoting diversity and inclusivity in the nursing profession as well as policies pointing to issues of violence to create a safer and more supportive learning environment for students.

Nurse educators on the other hand also have various experiences and challenges in their roles, as evidenced by several studies from different countries. These challenges include issues related to the education system, clinical learning, professionalism, violence, practical experiences, and adapting to changing circumstances like the COVID-19 pandemic. Preceptors and mentors play essential roles in facilitating students’ clinical teaching and learning, with more experienced preceptors having lower confidence in their role performance [47]. However, the mentorship and preceptorship practices are often informal, with unclear role expectations, resource challenges, and a lack of monitoring by nursing schools [46].

The issue of teaching strategies and students’ learning styles have also been addressed. The use of simulation-based education has been positively received in low-resource settings, with improved competence reported by nursing students [40]. However, nurse educators had not been exposed to and had limited experience utilizing high fidelity simulation (HFS) as a teaching method. Facilities equipped with HFS manikins are not used optimally due to ‘fear of the unknown’ experienced by the nurse educators because of their lack of training, and/or the lack of technological skills [32]. This underscores the effectiveness of innovative pedagogical methods in nursing education and preparation of nurse educators to undertake their roles.

### 5.3 Knowledge and attitude towards nursing education research

The study retrieved 7 studies that examined the knowledge and attitude towards nursing education researcg. Table 5 contains a summary of key findings regarding knowledge and attitude.

**Table 5:** Knowledge and attitude towards nursing education research (n = 7).

Study	Country	Study Design	Main Findings
[49]	Ghana	Descriptive cross-sectional	Educational interventions should be designed with consideration of both areas of strength and weakness. in pediatric pain knowledge and attitudes.
[50]	South Africa		undergraduate nursing student comprehensive understanding of HIV and AIDS is crucial for delivering high-quality care to people living with HIV/AIDS
[51]	Rwanda	Descriptive cross-sectional	Teaching evidence-based practice in the nursing school should be seamlessly integrated into the nursing curriculum across the entire undergraduate program, with a strong focus on both theoretical and practical aspects.
[52]	Nigeria	Descriptive cross sectional	Majority of final-year nursing students had insufficient knowledge about chronic kidney disease (CKD). To enhance patient management outcomes, it is imperative to expand the existing curriculum, providing these future nurses with a more comprehensive understanding of CKD’s fundamental concepts.
[53]	Ethiopia	Descriptive cross-sectional	Nurse educators generally have a favorable attitude toward clinical preceptorship, but there is a noticeable knowledge gap, with advanced degrees and teaching experience correlating with better knowledge.
[54]	Ethiopia	Descriptive cross-sectional	The main challenge in applying clinical simulation in nursing education, as identified by participants, was the lack of necessary materials.
[55]	Cameroon	cross-sectional descriptive	Nursing students exhibit slightly positive attitudes towards older people. To further enhance these attitudes, it is recommended to incorporate geriatric courses into nursing curricula.

African research in nursing education has explored the dimensions of knowledge and attitudes toward learning among nursing students from various angles. In response to identified deficiencies in knowledge or negative attitudes toward specific patient groups or conditions, there have been recommendations to incorporate certain topics into the nursing curriculum. These proposed curriculum enhancements encompass a range of areas, including gerontology [55], pediatric pain assessment [49], chronic kidney disease [52], and sexual health education and HIV and AIDS healthcare [50].

Furthermore, concerns have arisen regarding pedagogical approaches, particularly in the context of simulation-based education. The idea of integrating simulation into nursing training programs has been met with both enthusiasm and challenges. Some of these challenges relate to resource limitations and the already packed curriculum content [54].

In addition to these curriculum-related considerations, there has been a growing recognition of the evolving dynamics within the clinical environment. These changes have prompted calls to address knowledge gaps among nurse educators, particularly in areas such as preceptorship and clinical teaching [53]. Furthermore, there is a pressing need to bridge the theory-to-practice gap, emphasizing the importance of evidence-based practice throughout the curriculum [51]. In essence, research in the field of nursing education has shed light on the critical intersections of knowledge, attitudes, and pedagogy. These insights have informed recommendations for enhancing nursing curricula and teaching methodologies to better prepare future nurses for the complex and evolving healthcare landscape.

### 5.4 Perspectives of nurses on nursing education research

The review retrieved 15 studies that examined the perspectives of nurses on nursing education research. Table 6 contains a summary of key findings on their perspectives.

**Table 6:** Perspectives of nurses on nursing education research (n = 15).

Study	Country	Study Design	Main Findings
[56]	Uganda	Phenomenological	It is essential to elevate the initial qualification requirement for nursing to a bachelor’s degree level. Nevertheless, the number of BSN-trained nurses is limited, lacking motivation, and showing a preference for employment overseas rather than within Uganda. The creation of policies aimed at enhancing compensation and fostering a harmonious work environment is crucial to encourage the retention of these nursing professionals.
[57]	Malawi	Concurrent explorative descriptive mixed methods	There is a pressing need to improve clinical supervision and support for students. Nurse educators should develop effective plans for clinical supervision and support to foster the development of skilled nursing graduates.
[58]	South Africa	Phenomenological	Male nursing students, who are a minority in nursing education, face challenges related to professional stereotypes and discomfort in providing intimate care
[25]	Malawi	Hermeneutic phenomenological	Recognizing and understanding emotional labor in various contexts can contribute to the development of supportive and caring clinical learning environments for nursing students
[59]	South Africa	Multiple embedded case study	Nurse educators have a crucial role in the inclusion of student nurses with disabilities (SNWDs) in nursing education through integration, and advocating for early disclosure and support
[60]	Lesotho	Grounded theory	There is a shortage of simulation facilitators, which poses a challenge to the successful utilization of simulation. Additionally, students expressed apprehension about the feedback provided by educators during simulations, as some of the feedback appeared demeaning.
[61]	South Africa	Exploratory and descriptive	Even though the absence of spiritual care guidelines is an issue, Nurse Educators clearly demonstrated their readiness to instruct students in addressing patients’ spiritual needs. There’s a recognized requirement for a guiding theory and philosophy to establish a formalized approach to teaching spiritual care in nursing education.
[62]	Malawi	Phenomenological	Nursing and midwifery faculty exhibit a combination of caring and uncaring behaviors in their interactions with students. Promoting educational interactions between faculty and students that are grounded in moral and human caring principles enhance students’ professional development and well-being.
[63]	South Africa	Qualitative grounded theory	The integration of Public Nursing Colleges with higher education stemmed from political changes and the government’s commitment to improving the quality of nursing graduates. This improvement is expected to subsequently enhance the quality of healthcare service delivery in South Africa.
[31]	SSA-Madagascar, Tanzania	Descriptive phenomenological	Nurse educators generally view simulation as a valuable pedagogic tool for enhancing students’ learning experiences. However, the high number of students, packed timetable and a detailed curriculum posed practical difficulties.
[33]	South Africa	Case study	Training program intervention for nursing student-educators is effective in helping them achieve a balanced teacher identity that encompasses all three important dimensions: subject matter, pedagogical skills, and didactical expertise.
[64]	Ghana	Exploratory qualitative	Clinical nursing education can be enhanced through several measures; reducing student overcrowding in clinical settings, bridging the theory-practice gap, and ensuring the availability of relevant material resources in clinical facilities.
[65]	Rwanda	Literature review	Well-educated oncology nurses play a crucial role in caring for cancer patients and engaging in prevention activities.
[34]	South Africa	Descriptive, cross sectional	Nursing students face challenges related to IT training and internet connectivity. An IT module should be included in the nursing curriculum to enhance IT adoption and proficiency in nursing education.
67	Kenya	Phenomenological	Patient safety concepts should be seamlessly integrated into the nursing curriculum. Additionally, both academic and clinical faculties must be adequately equipped to effectively teach and assess these concepts.

The realm of nursing education research reveals a multifaceted landscape marked by a diverse array of perspectives and focal points. These insights, as documented in various studies, span a wide spectrum of critical areas within nursing education. One of the key areas of concern, as elucidated by [56], revolves around the pressing need to elevate the standards of nursing qualifications. This reflects an acknowledgment of the evolving demands within the healthcare landscape and underscores the necessity for nurses to attain a higher degree of educational preparation. Simultaneously, [57] shed light on the imperative of enhancing clinical supervision and support for nursing students. Their findings underscore the pivotal role played by effective clinical supervision in nurturing the growth of competent nursing graduates.

The work of [58] delves into the challenges faced by male nursing students, including navigating professional stereotypes and discomfort in providing intimate care. Emotional labor in various nursing contexts, as explored by [25] takes center stage in another dimension. Recognizing and comprehending emotional labor’s impact on nursing students can contribute to the development of nurturing and compassionate clinical learning environments. [59] bring to light the importance of inclusivity by advocating for the integration of students with disabilities in nursing education. Their work emphasizes the need for nursing educators to broaden their understanding of disability beyond the medical model.

Challenges in simulation education, as illuminated by [60], reflect the practical obstacles faced in incorporating simulation as an effective pedagogic tool. These challenges, including resource constraints and curriculum pressures, which need to be addressed to optimize simulation’s benefits. The significance of addressing IT-related challenges in nursing education, as emphasized by [34], lies in the pivotal role of technology in contemporary healthcare.

Spiritual care in nursing, as underscored by [61], emerges as a critical yet often overlooked aspect of nursing education. Their findings reveal a willingness among nurse educators to instruct students in addressing patients’ spiritual needs, signaling a need for formalized teaching approaches in this domain. Caring behaviors within the nursing faculty, as elucidated by [62], are essential for nurturing students’ professional development and well-being.

The integration of nursing colleges with higher education, as explored by [63], is influenced by political changes and a commitment to enhancing the quality of nursing graduates. This integration is expected to elevate the quality of healthcare service delivery in South Africa.

The pursuit of a balanced teacher identity for nursing student-educators, as advocated by [33], underscores the importance of equipping educators with a holistic blend of subject matter expertise, pedagogical skills, and didactical proficiency. Addressing challenges in clinical education, as discussed by [64], involves measures such as reducing student overcrowding, bridging the theory-practice gap, and ensuring the availability of relevant material resources in clinical facilities. [65] shed light on the establishment of oncology nursing programs as a critical step in addressing the specialized care needs of cancer patients. Their experience in Rwanda serves as a valuable reference for regions seeking to develop similar initiatives.

Finally, the seamless integration of patient safety concepts into the nursing curriculum, as advocated by [66], encompasses several facets. This includes curriculum integration, equipping faculty for effective teaching and assessment, fostering a patient safety culture in clinical settings, and enhancing collaboration between academic and clinical environments. Collectively, these findings illuminate the dynamic and evolving landscape of nursing education in Sub-Saharan Africa. They highlight not only the challenges but also the opportunities for elevating the quality of nursing education and, consequently, enhancing the caliber of healthcare service delivery within the region. Integrating these insights can contribute significantly to the enhancement of nursing education programs, better preparing future nursing professionals to meet the evolving healthcare demands of their respective countries.

## 6. Policy recommendations

Simulation based education: Government and institutions should invest in simulation infrastructure by allocating dedicated funding for simulation labs, including high-fidelity manikins and virtual simulation platforms. Additionally they should establish national or regional simulation training centers to upskill nursing educators in simulation-based teaching methodologies

Regulatory bodies should mandate a minimum percentage of clinical training hours through simulation-based learning in nursing curricula.

Inclusion of technology in learning: Governments should ensure reliable internet connectivity and access to digital learning platforms for all nursing education institutions.They should as well establish continuous faculty development programs on integrating technology into teaching, including e-learning tools and virtual patient simulations. Subsidies or low-cost loan programs for nursing students to acquire necessary digital devices such as tablets or laptops should be instituted. Nursing curricula should incorporate AI-driven tutoring systems, chatbots, and adaptive learning platforms to personalize student learning experiences and enhance competency development.

## 7. Conclusion

There is dearth of literature from SSA on nursing education that touches on experiences of nurse educators and nursing students. In this systematic review, we have examined a diverse range of studies conducted in different countries to understand the experiences and challenges faced by nursing and midwifery students during their education and clinical training. These experiences cut across classroom teaching, clinical learning environment and clinical practice. The studies encompassed a variety of research designs, from qualitative exploratory to descriptive quantitative, and have shed light on key themes and findings related to the students’ educational journey. Nursing students and new graduated nurses across SSA share almost the same experiences in the clinical areas.

The bibliometric analysis aspect of the study reveals that nursing education has evolved in SSA since its inception. The systematic review approach of the research adds to the body of knowledge and enhances efforts of stakeholders in the improvement of the quality, quantity and relevance of nursing education in Sub Saharan Africa.

This study reveals the multifaceted experiences and challenges faced by nursing and midwifery students in diverse settings. Educational support, effective communication, professionalism, and inclusivity are crucial aspects that require attention and improvement. Innovative teaching methods, such as simulation-based education, have shown promise in enhancing students’ competence but not without good preparation of nurse educators.

These findings of this review provide valuable insights for educators, policymakers, and institutions to enhance the quality of nursing education and ensure that students are well-prepared to meet the demands of the healthcare profession.

One of the limitations of this research is that it only considered nursing education research in Sub-Saharan Africa. This means that the findings of the research may not be applicable to other regional contexts as SSA face a myriad of challenges. Future research can be undertaken to examine nursing education research trends in other regions, expecially in Low- and Middle-income Countries.
